# The effect of planning time on vocabulary acquisition in a task-based environment: the mediating roles of working memory and field (in)dependence

**DOI:** 10.1186/s40359-024-01638-4

**Published:** 2024-03-13

**Authors:** Jingjing Gui, Sayed M. Ismail

**Affiliations:** 1 School of Automotive Engineering, Guangdong Polytechnic of Industry and Commerce, 510510 Guangdong Province, China; 2 Department of English Language, College of Sciences and Humanities, Prince Sattam bin Abdulaziz University, Al-kharj, Saudi Arabia

**Keywords:** Field dependence, Field independence, Planning time, Task-based language teaching, Vocabulary learning, Working memory

## Abstract

This research investigated the impact of planning time, working memory (WM), and cognitive styles on language learning outcomes within the framework of Task-Based Language Teaching (TBLT). Drawing on a diverse sample of language learners, the study employed a pretest-posttest control group quasi-experiment to examine the effects of providing pre-task planning time on language performance. Participants engaged in communicative tasks, with a focus on vocabulary acquisition and task complexity, while their cognitive processes were assessed through measures of WM and cognitive styles. The findings revealed significant interactions between planning time and cognitive styles, particularly field dependence, influencing language production and proficiency such that learners with planning time outperformed learners without planning time; high-WM learners outperformed their low-WM peers, and field independent learners outstripped their field-dependent counterparts. Moreover, the study contributes to the broader understanding of the nuanced relationship between planning time, WM, and cognitive styles in the context of TBLT. The implications of these findings for language teachers, materials developers, syllabus designers, curriculum developers, and policymakers are discussed, offering insights into the design of effective language learning environments. Despite certain limitations, the study provides a foundation for further research exploring cross-cultural variations, longitudinal effects, and the integration of technology in language education, with the aim of advancing pedagogical practices tailored to diverse learner profiles.

## Introduction

In the dynamic landscape of language acquisition research, understanding the multifaceted interactions that shape vocabulary acquisition is essential for unraveling the complexities of effective language learning strategies [1]. This study delves into the intriguing nexus between planning time, a crucial aspect of TBLT, and vocabulary acquisition. As language educators and researchers alike grapple with optimizing instructional approaches, the role of cognitive mechanisms, such as WM, and individual cognitive styles, such as field (in)dependence (FI/D), emerge as potential mediating factors in this intricate process [2].

Effective communication stands at the core of the human experience, encompassing vital processes such as listening, speaking, reading, and writing. Proficiency in these four language skills holds great significance for students aiming to engage with a global audience. The mastery of these skills is paramount for individuals who aspire to effectively communicate and articulate their ideas, emotions, and opinions. Several factors influence the mastery of language skills, with vocabulary and grammar emerging as key components [3]. emphasizes the indispensability of vocabulary, asserting that conveying any message hinges on it. This underscores the pivotal role vocabulary plays, particularly in the context of learning English. The quality of an individual’s language is intricately tied to the richness of their vocabulary, as noted by [4]. They posit that vocabulary serves as a fundamental element of language proficiency, shaping how learners express themselves. Consequently, the role of vocabulary in language is deemed crucial, serving as a conduit for ideas both in written and oral communication. Understanding vocabulary as a supporting pillar of language skills is imperative, given its profound impact on the meaning and message an individual seeks to convey [5].

Building on this, in the realm of language education, TBLT emerges as a dynamic pedagogical approach designed to immerse learners in authentic and purposeful language use [6]. TBLT, as a departure from traditional methods, emphasizes the integration of language skills through the completion of meaningful tasks. These tasks, ranging from problem-solving activities to collaborative projects, not only contextualize language learning but also offer a fertile ground for vocabulary acquisition [7]. By engaging learners in tasks that mirror real-world communication demands, TBLT encourages the active application of vocabulary within relevant contexts, fostering a deeper and more nuanced understanding of language use [8].

Moreover, the interactive nature of task-based activities promotes the utilization of a broad spectrum of vocabulary, encouraging learners to explore and internalize words in diverse scenarios. Furthermore, the inherent focus on communication goals in TBLT aligns with the communicative nature of language, reinforcing the practical utility of acquired vocabulary in conveying ideas and achieving specific language objectives [8]. As learners navigate these tasks, planning time becomes a critical element in shaping the depth and precision of their language production.

In addition, in the context of language learning and task-based activities, planning time refers to the dedicated period given to learners before engaging in a communicative task. This preparatory phase allows individuals to strategize, organize thoughts, and select appropriate vocabulary and language structures to effectively convey their intended message [9]. Planning time serves as a cognitive rehearsal, enabling learners to anticipate challenges, formulate responses, and tailor their language production to meet the communicative goals of the task at hand.

Research indicates that the provision of planning time significantly influences language performance by allowing learners to engage in metacognitive processes [10]. During this temporal interval, individuals can activate their WM, a cognitive system crucial for the temporary storage and manipulation of information, to plan and structure their language output effectively [11]. This intentional cognitive effort not only enhances the accuracy and complexity of the language produced but also contributes to the language learning process [12].

Furthermore, planning time aligns with the principles of TBLT, which emphasizes the importance of strategic thinking and goal-oriented communication. By integrating planning time into language tasks, educators provide learners with a scaffolded approach to language production, allowing them to navigate the intricacies of vocabulary selection and application within the parameters of the given task [13].

As an individual difference train, WM, a fundamental cognitive system, plays a pivotal role in the intricate process of vocabulary acquisition. WM refers to the cognitive capacity responsible for temporarily holding and manipulating information during complex cognitive tasks [14]. In the context of language learning, WM serves as a mental workspace where learners can actively process and store linguistic elements, including vocabulary, grammar rules, and sentence structures [15]. The limited capacity of WM necessitates efficient cognitive processes, making it a critical component in the acquisition and retention of new lexical items.

When engaged in vocabulary learning, WM acts as a dynamic cognitive mechanism that facilitates the encoding, rehearsal, and retrieval of linguistic information [14]. Learners utilize WM to link new words to existing knowledge, form associations, and establish connections that aid in the storage and retrieval of vocabulary items during language production. The active engagement of WM in these processes not only strengthens the neural networks associated with vocabulary but also enhances the efficiency of language recall [15].

Moreover, the interplay between WM and vocabulary acquisition is particularly pronounced in TBLT settings. As learners navigate communicative tasks, WM becomes a cognitive scaffold, enabling them to manipulate linguistic elements, such as selecting appropriate vocabulary, constructing grammatically sound sentences, and adapting language use to the communicative context [11].

As another individual difference trait, FI/D represents an individual’s cognitive style characterized by the degree to which they rely on contextual cues in information processing. Those classified as field-independent (FI) tend to separate details from the surrounding context, focusing on discrete elements, while field-dependent (FD) individuals perceive information holistically, considering the broader context (16). In the realm of language learning, an individual’s field (in)dependence (FI/D) can significantly impact how they approach and comprehend linguistic input.

FI learners may excel in isolating and analyzing specific components of language, allowing for meticulous attention to vocabulary, grammar rules, and syntactic structures [16]. This cognitive style may lead to a heightened ability to discern nuanced meanings and relationships among words, potentially facilitating vocabulary acquisition through a more analytical and detail-oriented approach. On the other hand, FD learners, with their holistic processing preference, may excel in grasping the overall meaning and communicative intent of language, integrating vocabulary within a broader context [16]. This approach may contribute to a more intuitive and contextualized understanding of vocabulary use in real-life communication.

Despite the acknowledged importance of vocabulary acquisition in language learning, there exists an underexplored domain pertaining to the intricate interplay of planning time, WM, and FI/D within the context of TBLT. While TBLT is recognized for its dynamic and immersive approach to language instruction, the precise mechanisms by which planning time shapes vocabulary acquisition within this pedagogical framework, along with the mediating roles of WM and individual cognitive styles, remain incompletely understood. This research aims to untangle these complexities, offering nuanced insights to theoretical frameworks in language acquisition and providing practical applications for educators. The study addresses the following research questions to bridge this knowledge gap:

1. How does planning time affect the acquisition of vocabulary in a task-based environment?
2. How does WM affect the acquisition of vocabulary in a task-based environment?
3. How does field (in)dependence affect the acquisition of vocabulary in a task-based environment?

Through the exploration of these questions, the research strives to enhance our understanding of effective vocabulary learning strategies in TBLT, thereby contributing valuable guidance for educators and researchers in language education.

This study can hold paramount significance in the dynamic landscape of language education and TBLT by delving into the intricate dynamics of vocabulary acquisition. As language proficiency, particularly in English, is intricately tied to the richness of vocabulary, understanding the nuanced interactions between planning time, WM, and individual cognitive styles (FI/D) within the context of TBLT is crucial. The significance of this research is threefold:

1. **Informing Pedagogical Practices**: The findings of this study have the potential to inform and optimize pedagogical practices, especially within the framework of TBLT. Educators will benefit from insights into how planning time, WM, and cognitive styles contribute to vocabulary acquisition, allowing them to tailor instructional strategies for more effective language learning outcomes.
2. **Enhancing Language Learning Strategies**: By unraveling the specific mechanisms through which planning time influences vocabulary acquisition, this study contributes to the enhancement of language learning strategies. The identification of the mediating roles of WM and FI/D offers a nuanced understanding of the cognitive processes involved in acquiring and retaining new lexical items.
3. **Contributing to Theoretical Frameworks**: The research addresses a significant gap in the theoretical frameworks of language acquisition, particularly in the context of TBLT. The exploration of planning time, WM, and FI/D as factors influencing vocabulary acquisition enriches the theoretical understanding of how cognitive processes intersect in language learning.

Moreover, the research questions posed– examining the effects of planning time, WM, and FI/D on vocabulary acquisition in a task-based environment– pave the way for empirical investigations that bridge theory and practice. The outcomes of this study are poised to benefit language educators, researchers, and policymakers seeking evidence-based insights to enhance language learning methodologies and curricular design.

## Literature review

### Theoretical framework

#### Task-based language teaching

The term TBLT denotes an instructional approach initially implemented in the 1980s, garnering significant attention and advancement in second language teaching and acquisition. As per [17], TBLT is an instructional strategy that involves assigning students communicative tasks and fostering idea-sharing to attain desired outcomes. Consequently, TBLT significantly influences the development of communicative proficiency. According to Mckinnon and Rigby cited in [17], when educators render language relevant and comprehensible to students in the classroom, learners naturally absorb it. The planning and execution of TBLT encompass numerous tasks, identified as pivotal components in language education by [18]. In contrast to activities focused on form, the authors assert that learners’ participation in task-based endeavors creates a more advantageous environment for initiating learning processes, ultimately providing superior opportunities for language learning to unfold. The tasks in TBLT mirror real-life scenarios, underscoring the paramount importance of these activities.

Various scholars and educators provide distinct definitions for tasks. Long (cited in [19]) characterizes tasks as specific assignments undertaken for personal benefit or the advantage of others, either without compensation or in exchange for a benefit [8]. ‘s definition contributes to a clearer understanding of the term. Tasks are classified into two syllabus-based categories: pedagogic tasks and real-world tasks. Pedagogic tasks encompass a range of activities or exercises performed in the classroom, while real-world tasks pertain to activities learners might encounter in authentic situations [20]. defines tasks as activities that necessitate learners to communicate in the target language to achieve a specific objective. To successfully complete tasks within the given timeframe, with meaningful understanding and in the target language, learners must grasp their objectives. These tasks primarily aim for communication, thus minimizing attention to grammar or structural elements in the completion process. Therefore, a task in English reading classes can be delineated as a reading activity wherein students comprehend the meaning, modify the language used in the text, and produce output in the target language with the assistance of teachers and peers.

Reference [21] formulated a TBLT framework organized into three stages: (1) pre-task, offering a task overview, (2) task cycle, encompassing the task, planning, and report phases, and (3) language focus, involving analysis and practice.

The pre-task phase aims to introduce and delineate the topic. The instructor’s role involves aiding learners in recalling their existing knowledge about the subject, fostering extensive engagement in brainstorming exercises [7]. There are instances where teachers may invest substantial preparation in guiding students through the introduction of related-topic terms or phrases, identifying those words in the text, or facilitating collaborative efforts. The task cycle stage comprises three elements: task, planning, and report. Learners collaborate in pairs or groups during the task phase, with the teacher serving as a facilitator [8]. Planning involves students outlining their reports, with teacher guidance provided as needed. In the report phase, students present their work in the target language, while the teacher listens and offers feedback. The language focus stage allows for a comprehensive examination of specific language features employed during the task cycle [9]. In essence, after prioritizing meaning, the teacher guides the class to shift attention to language use and form that will prove beneficial in the future.

#### Planning time

The concept of planning time in the context of language acquisition finds its theoretical roots in cognitive and psycholinguistic frameworks. Cognitive models, such as the Information Processing Model (IPM) and the Cognitive Resource Theory (CRT), offer foundational perspectives on the role of planning time in language tasks. According to these models, planning time is viewed as a crucial phase that allows learners to engage in metacognitive processes, allocating cognitive resources strategically to enhance the quality of language production [21]. The IPM posits that effective language performance involves a series of cognitive processes, and planning time serves as a dedicated interval during which learners can organize their thoughts, select appropriate vocabulary, and plan the structure of their linguistic output [22].

Additionally, the cognitive resource theory suggests that the availability of sufficient planning time contributes to optimizing cognitive resources during language tasks. This theoretical framework contends that learners engage in systematic cognitive activities, such as rehearsal and organization, during the planning phase, which, in turn, supports the effective use of WM and facilitates the integration of new vocabulary into language production [23]. Planning time, within the context of these cognitive models, is conceptualized as a cognitive rehearsal mechanism that enables learners to enhance the accuracy and complexity of their language output.

From a psycholinguistic perspective, the theoretical underpinnings of planning time draw on theories of language production, including Levelt’s model of speech production. According to Levelt, speech production involves distinct stages, including conceptualization, formulation, and articulation [24]. The planning time aligns with the formulation stage, allowing learners to transform abstract concepts into linguistic structures and select appropriate lexical items. The theoretical framework of speech production emphasizes the dynamic interplay between planning time and the formulation of linguistic structures, shedding light on how this temporal interval influences vocabulary selection and arrangement in language tasks [25].

#### Working memory

WM, a pivotal construct in cognitive psychology, refers to the temporary storage and manipulation of information essential for ongoing cognitive tasks [22]. Unlike long-term memory, which involves the storage of information over extended periods, WM is transient and plays a crucial role in immediate cognitive processes. Baddeley’s influential model conceptualizes WM as comprising multiple components, with the phonological loop being particularly pertinent to language processing. The phonological loop is responsible for the temporary storage of auditory information and its rehearsal, making it vital for tasks involving language comprehension and production [26].

In the context of language acquisition, WM serves as a dynamic cognitive system facilitating various aspects of language processing. One fundamental role is in vocabulary acquisition. As learners encounter new words, WM enables the temporary storage of these lexical items, allowing for their integration into the mental lexicon [1]. Furthermore, WM is engaged during language production, aiding in the selection and arrangement of words within sentences [11]. For example, when constructing a sentence, individuals actively hold the words they plan to use in their WM, facilitating the syntactic and semantic processes required for coherent expression.

The influence of WM on language learning is multifaceted. As learners engage with linguistic input, WM is actively involved in the processing and integration of new information. For instance, during the initial stages of vocabulary acquisition, WM helps link new words to existing knowledge, forming associations that enhance retention [27]. Additionally, WM is instrumental in sentence comprehension, allowing individuals to parse and interpret complex syntactic structures by temporarily holding and manipulating linguistic elements.

Theoretical perspectives, such as Baddeley’s model, highlight the intricate interplay between WM and language processing. The limited capacity of WM necessitates efficient cognitive processes during language tasks, making it a critical component in the acquisition and retention of new vocabulary [28]. Moreover, WM is integral to the execution of higher-order cognitive functions involved in language learning, such as problem-solving, comprehension, and production.

#### Field (in)dependence

Field independence, a cognitive style identified by Witkin and colleagues, refers to an individual’s tendency to perceive and process information independently of its surrounding context [29]. This cognitive style is contrasted with field dependence, where individuals rely on the context and external cues in information processing. The concept of field independence is grounded in Witkin’s theory of cognitive styles, which posits that individuals exhibit consistent and enduring preferences in how they approach and organize information.

The theoretical underpinnings of field independence are rooted in the assumption that individuals possess distinctive cognitive structures influencing their perceptual and cognitive processes. Witkin proposed that FI individuals have a natural inclination to focus on discrete elements within a perceptual field, segregating them from the context. This analytical approach allows for detailed and systematic processing of information, emphasizing the separation of parts from the whole [29].

Field independence has been associated with various cognitive processes, including problem-solving, learning strategies, and information processing in different domains. In the context of language learning, FD learners may excel in isolating and analyzing specific components of language, such as vocabulary, grammar rules, and syntactic structures [30]. This cognitive style suggests a preference for an analytical and detail-oriented approach to language tasks, potentially influencing how individuals acquire and process linguistic information.

Research has explored the impact of field independence on language learning outcomes. For example, FI learners may exhibit strengths in tasks that require attention to detail, such as grammar exercises and vocabulary memorization [30]. However, the relationship between field independence and language learning is complex, and individual differences play a significant role.

### Empirical background

#### TBLT and language learning

Language instruction, especially English for specific purposes (ESP) with Computer-Assisted Language Learning (CALL), has gained popularity. TBLT enhances CALL effectiveness [31]. compared Computer-Assisted English Lessons for Logistics using TBLT with traditional instruction. The 48 Thai EFL college learners were randomly assigned to the control (*N* = 24) and experimental groups (*N* = 24). The experimental group received CALL-based instruction with TBLT, while the control group had traditional instruction. The one-way MANOVA results showed the experimental group significantly outperformed the control group in both receptive and productive vocabulary posttests.

Reference [32] aimed to assess the impact of technology-mediated TBLT on descriptive writing in a Thai EFL context, using the FlipGrid application. The study involved 29 2nd-year university students and employed a mixed-methods approach. Results showed significant improvements in various writing aspects, with “grammatical range and accuracy” exhibiting the most progress. Students found the approach innovative and learner-centered, fostering collaboration and effective descriptive writing.

To address passive vocabulary learning issues among Chinese EFL students, TBLT methods were implemented in university English classes. In TBLT, students engaged in English interactions, utilizing learned words for teacher-designed tasks, grounded in second language acquisition theories. The study, conducted by [33], focused on incorporating TBLT in English vocabulary teaching across three non-English major classes at a Chinese university, emphasizing the use of word games. An online questionnaire surveyed 93 university students, and an analysis of before-task and after-task word quizzes on the Moodle platform, along with exam scores, revealed that students enjoyed word games, benefiting language development and skill acquisition.

Providing pre-task planning time for second language speech tasks shows potential in enhancing focus on form alongside meaning-focused instruction. In [34], the impact of planning time on task-based oral performance in EFL learners was examined. The study involved 52 Saudi high school students from Riyadh, randomly assigned to experimental and control groups. The participants performed the task with five minutes of pre-task planning and without any planning time, respectively. Recorded and transcribed performances were assessed for fluency, accuracy, and complexity. T-test results demonstrated that participants with pre-task planning significantly outperformed those without planning. Consequently, this study suggests that learners, when granted pre-task planning time, can produce language that is more fluent, accurate, and complex compared to those starting the task immediately. Additionally, there was no observed trade-off competition among fluency, accuracy, and complexity.

Research on pretask planning has typically focused on its impact on fluency, accuracy, and complexity, neglecting its influence on broader discourse elements. Addressing this gap, study [35] explored the effects of pretask planning on Chinese EFL learners’ selection of referential expressions in oral narratives. Fifty-six intermediate-level learners retold the story of Modern Times with either 10 min of strategic planning or no planning. An additional 25 native speakers performed the same task. Analysis revealed that learners tended to be overly explicit in singular character reference, and pretask planning improved target-like expressions for major characters, moderated by role prominence.

Research in TBLT has explored how planning influences task performance, yet scant attention has been given to the intricacies of the planning processes themselves [36]. extended prior research by offering a detailed examination of collaborative pre-task planning by four Japanese university learners (two dyads). Their subsequent performance on an L2 oral monologue task, requiring them to express opinions and propose solutions, was also analyzed. Follow-up interviews with stimulated recall provided additional insights. The findings highlighted variations in planning processes and task performances due to note-taking strategies, interpersonal dynamics, L2 proficiency, and the choice of language (L1 or L2) for planning.

#### WM and language learning

Efficient WM boosts cognitive abilities during multimedia learning. In [37], the role of WM in vocabulary learning through multimedia input was explored, examining associations between executive WM, phonological short-term memory (PSTM), and three input conditions (Definition + Word info + Video, Definition + Word info, and Definition). Ninety-five students engaged in learning sessions under these conditions, completing reading span and non-word span tests. Results, analyzed through repeated-measures ANCOVA, emphasized the impactful Definition + Word info + Video condition on vocabulary learning and retention. Complex and phonological WM significantly contributed to vocabulary learning across all conditions.

With the objective of enhancing our comprehension of how task complexity and learner-internal factors influence L2 performance, a 2 × 2 within-between participant study was crafted [38]. aimed to investigate the impact of intentional reasoning on L2 performance and whether language proficiency and WM mediated these effects. Forty-eight English learners engaged in two video-based narrative tasks, each varying in intentional reasoning, subsequent to undertaking the Oxford Placement Test, Elicited Imitation Tasks, and backward-digit span tasks. Findings revealed significant effects of intentional reasoning on complexity and accuracy, with no influence on fluency. Regression analyses highlighted the predictive roles of proficiency and WM on accuracy across both task types. However, their contributions to models predicting lexical complexity and fluency speed varied, emphasizing the intricate interplay between task complexity and learner-internal factors.

Reference [39] investigated incidental vocabulary learning from captioned videos across genres for 210 EFL learners. They explored the impact of video types (comedy, education, and documentary), repetition (once and twice), and WM. Results favored the comedy genre, with repeated viewing showing significance in immediate form recognition. Complex WM impacted delayed meaning recognition and recall. Vocabulary knowledge and comprehension significantly influenced incidental vocabulary learning.

#### FI/D and language learning

Cognitive styles, as individual differences among learners, play a pivotal role in predicting learning behavior. This emphasizes their significance in the design of online learning. Various cognitive style dimensions exist, and overlaps among these dimensions are evident. Notably, Witkin’s FI/D and Pask’s Holism/Serialism exhibit similarities. Consequently, there is a need to create a framework illustrating the overlapped behavior between these two cognitive style dimensions. In addressing this, [40] utilized Lag Sequential Analysis to scrutinize the overlaps in the context of online learning behavior. The study’s findings indicated that these overlaps are prominent in comprehensive/local and dynamic/fixed approaches.

Reference [41] presents findings from a classroom-based inquiry examining the influence of field independence on the efficacy of processing instruction. It involved fifty-six students from a high school in Iran, randomly assigned to an experimental group exposed to processing instruction and a comparison group receiving traditional output-based instruction on English passive. A pretest-treatment posttest design, employing a sentence-level interpretation task, gauged instructional impact. The outcomes revealed a significant enhancement in the acquisition of the target structure through processing instruction, contrasting with the ineffective traditional instruction. Importantly, no interaction surfaced between learners’ field independence and their performance in both immediate and delayed posttests.

The studies collectively contribute valuable insights into language instruction and learning, particularly within the framework of TBLT and CALL. In [31], the integration of CALL with TBLT for English lessons resulted in significantly improved vocabulary outcomes compared to traditional instruction. Similarly, [32] explored the use of technology-mediated TBLT, specifically with the FlipGrid application, leading to enhanced descriptive writing skills among university students. TBLT methods were also employed in [33] to address passive vocabulary learning issues among Chinese EFL students, demonstrating positive outcomes through word games. The impact of pre-task planning on oral performance was investigated in [34], revealing that learners with planning time produced more fluent, accurate, and complex language [35]. focused on pretask planning’s influence on referential expressions in oral narratives, emphasizing improvements with strategic planning. The intricacies of collaborative pre-task planning were explored in [36], providing insights into variations influenced by note-taking, interpersonal dynamics, and language choice. WM’s role in vocabulary learning through multimedia input was studied in [37], emphasizing the significance of executive and phonological WM. Task complexity and learner-internal factors were examined in [38], highlighting the effects of intentional reasoning on L2 performance, mediated by proficiency and WM [39]. delved into incidental vocabulary learning from captioned videos, considering video types and repetition, with implications for comprehension and recall. Cognitive styles, specifically field independence, were investigated in [40], revealing overlaps with Pask’s Holism/Serialism in the context of online learning behavior. Finally, [41] demonstrated the effectiveness of processing instruction, independent of learners’ field independence, in enhancing the acquisition of the English passive structure. These studies collectively contribute to a nuanced understanding of language learning dynamics, offering implications for pedagogical practices and theoretical frameworks.

Given the extensive exploration of TBLT, planning time, WM, and cognitive styles in the literature, it is evident that these components significantly impact language learning outcomes. While TBLT has been recognized for its effectiveness in enhancing communicative proficiency and providing real-world language learning experiences, the specific contributions of planning time, WM, and cognitive styles need further examination. The role of planning time in facilitating metacognitive processes and its impact on language production remain crucial aspects to explore. Additionally, understanding how WM influences vocabulary acquisition and language processing during tasks is essential for optimizing language learning strategies. Furthermore, the cognitive style of field independence, with its emphasis on analytical processing, requires a deeper investigation to discern its implications for language learning outcomes. Integrating these insights into language instruction, especially within the realm of online learning, can lead to more tailored and effective language teaching methodologies. Thus, this study aims to investigate the nuanced interplay of TBLT, planning time, WM, and cognitive styles to provide comprehensive insights for educators and researchers in the field of second language acquisition.

## Method

### Design

This study employs a quantitative quasi-experimental pretest-posttest design with a control group to investigate the influence of planning time on vocabulary acquisition within the TBLT framework. The research design allows for the systematic examination of the effects of planning time on vocabulary learning outcomes by comparing the performance of learners who receive dedicated planning time before engaging in communicative tasks to a control group without such intervention. This robust design enables the exploration of causal relationships and the identification of potential mediating factors, such as WM and FI/D, contributing to a comprehensive understanding of the intricate dynamics at play in the language learning process.

### Setting and participants

The study was conducted in Applied College of Prince Sattam Bin Abdulaziz University, involving three intact classes with a total of 54 participants. Each class consisted of 18 learners who were randomly assigned to three distinct conditions: an experimental group (EG) with dedicated planning time before communicative tasks, another EG without planning time, and a control group (CG). We divided the learners into these distinctive groups to measure the exact effect of planning time on learners’ vocabulary gains both with and without TBLT. All participants shared Arabic as their native language and had undergone English language instruction for seven years before their involvement in the study. The age range of the learners was between 14 and 16 years, and none of them had previously visited an English-speaking country. This carefully selected participant pool aimed to provide insights into the effects of planning time on vocabulary acquisition within the specific context of Saudi Arabian English language learners with a homogeneous linguistic background and similar educational experiences.

### Instruments

The study employed various instruments to assess different aspects of the participants’ language skills and cognitive abilities. The primary instructional material was Life by Helen Stephenson, serving as the basis for vocabulary instruction. To evaluate the learners’ pre-existing knowledge of vocabulary, a vocabulary test was developed by (5), and the known-group technique [42] was utilized for validation, that is we administered the test to a panel of language teachers whose performance differed significantly from those of our participants at the outset of the study (*p* = 0.001), hence the construct validity of our instrument. Additionally, using the KR-21 formula, the test’s reliability was shown to be high (*r* = 0.87). This same test, presented in a different format, was administered as the posttest to gauge changes in vocabulary knowledge over the course of the study. In addition to vocabulary assessment, the study utilized a reading-span test, developed by [43], to measure the WM of the participants. The reading-span test is a well-established tool for evaluating the cognitive capacity responsible for the temporary storage and manipulation of information during complex cognitive tasks. Furthermore, the study employed a group-embedded figures test (GEFT), developed by [44], to measure the participants’ FI/D. This instrument assessed the degree to which learners rely on contextual cues in information processing, providing insights into their cognitive styles during language tasks.

### Data collection procedures

The study implemented a quantitative quasi-experimental design with a pretest-posttest control group to investigate the impact of planning time on vocabulary acquisition, considering the mediating roles of WM and FI/D. The participants, 54 learners aged 14 to 16, were drawn from three intact classes in Applied College, Prince Sattam Bin Abdulaziz University, Saudi Arabia. These classes were randomly assigned to three conditions: one experimental group with planning time, another experimental group without planning time, and a control group.

The instruments used for data collection included the book “Life” by Helen Stephenson as the instructional material, a teacher-made vocabulary test for pretest and posttest assessments, a reading-span test to measure WM, and a GEFT for evaluating FI/D.

In the treatment phase of the study, the participants were divided into three groups: an EG with planning time (EG1), an EG without planning time (EG2), and a CG. The treatment involved the use of the Oxford Word Skills textbook (2nd edition) as instructional material for all three groups.

For EG1, learners were provided with a dedicated planning time before engaging in communicative tasks. This planning phase allowed them to strategize, organize their thoughts, and select appropriate vocabulary and language structures for the subsequent task. This intentional cognitive effort during planning aimed to enhance the accuracy and complexity of language production, as well as contribute to the retention and application of newly acquired vocabulary.

EG2, the second experimental group, participated in communicative tasks without any specific planning time. This group aimed to provide insights into the impact of immediate task engagement without the benefit of a dedicated planning phase.

The communicative tasks designed for this study were meticulously crafted to mirror real-world scenarios, ensuring alignment with the principles of TBLT and the simulation of authentic language production. In the context of TBLT, tasks were purposefully selected to engage participants in meaningful and goal-oriented communication, promoting the application of language skills within relevant and authentic contexts. For instance, a task involved participants planning and executing a collaborative problem-solving activity, akin to a real-world scenario where individuals must work together to achieve a common objective. Another task required learners to engage in a simulated business negotiation, encouraging the use of specialized vocabulary and language structures in a context reminiscent of professional interactions. By incorporating tasks that parallel genuine communicative situations, the study aimed to capture a more holistic and practical assessment of language learning, allowing participants to apply their vocabulary acquisition within scenarios that reflect the complexity of real-world language use.

The CG, on the other hand, received a teacher-fronted treatment and followed their usual instructional routine. This group did not engage in specific communicative tasks designed for the study, serving as a baseline comparison to assess the effects of planning time on vocabulary acquisition in the EGs.

It is important to note that the textbook used in this study was consistent across all conditions, ensuring uniformity in instructional materials. All participants, regardless of their assigned experimental group or control group, utilized the same textbook as the primary resource for language learning. This uniformity in learning materials aimed to eliminate potential confounding variables related to instructional content, allowing a more focused examination of the distinct influences of planning time, working memory, and cognitive styles on vocabulary acquisition within the task-based language teaching framework.

The communicative tasks were designed to reflect real-world scenarios, aligning with the principles of TBLT. Planning time for EG1 was incorporated to simulate authentic language production scenarios, emphasizing the importance of strategic thinking and goal-oriented communication. The differences in treatment among the three groups allowed for a nuanced exploration of the role of planning time in vocabulary acquisition.

### Data analysis procedures

To analyze the data and assess the impact of planning time on vocabulary acquisition, one-way ANOVA tests were conducted for each of the two-time intervals. This statistical method allowed for a comparison of means across the three groups—EG1, EG2, and the control group.

Furthermore, to investigate the influence of WM on the vocabulary acquisition process, separate ANOVA tests were conducted for each time interval. This analysis aimed to explore the potential relationship between WM capacity and vocabulary acquisition outcomes.

In addition, to examine the effect of FI/D, ANOVA tests were conducted for each time interval. This statistical approach facilitated the exploration of how individual cognitive styles, specifically FI/D, might contribute to variations in vocabulary acquisition across the three groups.

These ANOVA tests provided a robust statistical framework to compare the means and evaluate the significance of differences in vocabulary acquisition scores, WM impact, and FI/D effects within the defined time intervals. The chosen analyses allowed for a comprehensive understanding of the relationships between planning time, WM, cognitive styles, and vocabulary acquisition in the study context.

## Results

### The effect of planning time on vocabulary acquisition

As mentioned in the preceding section, to study planning time’s impact on vocabulary growth, we conducted an ANOVA for each time interval. However, to ensure the data normality, a Kolmogorov-Smirnov (K-S) test was conducted whose results are presented below.

**Table 1:** One-sample Kolmogorov-Smirnov test of vocabulary learning

		Pretest scores	Posttest scores
N		54	54
Normal parameters	Mean	3.481	8.925
	Std. Deviation	1.819	5.305
Most extreme differences	Absolute	0.126	0.182
	Positive	0.126	0.135
	Negative	− 0.121	− 0.182
Kolmogorov-Smirnov Z		0.923	1.336
Asymp. Sig. (2-tailed)		0.362	0.056

Table 1 indicates that on both time intervals, the data are normally distributed since the p-value is greater than 0.05.

**Table 2:** Descriptive statistics of planning time’s effect on vocabulary learning on the pretest

Group	Mean	Std. Deviation	N
EG1	3.333	1.878	18
EG2	3.777	1.733	18
CG	3.333	1.909	18
Total	3.481	1.819	54

Table 2 indicates that on the pretest EG1 (M = 3.333, SD = 1.878), EG2 (M = 3.777, SD = 1.733), and the CG (M = 3.333, SD = 1.909) performed exactly the same.

**Table 3:** Tests of between-subjects effects of planning time’s effect on vocabulary learning on the pretest

Source	Type III sum of squares	df	Mean square	F	Sig.	Partial eta squared
Corrected model	2.370	2	1.185	0.349	0.707	0.014
Intercept	654.519	1	654.519	192.827	0.000	0.791
Group	2.370	2	1.185	0.349	0.707	0.014
Error	173.111	51	3.394			
Total	830.000	54				
Corrected total	175.481	53				

Table 3 indicates that groups’ difference was not significant on the pretest (df = 2, F = 192.827, *p* = 0.707).

**Table 4:** Descriptive statistics of planning time’s effect on vocabulary learning on the posttest

Group	Mean	Std. Deviation	N
EG1	14.1667	1.85504	18
EG2	9.2778	4.37648	18
CG	3.6333	1.68034	18
Total	8.9259	5.30522	54

Based on Table 4, on the posttest, EG1 (M = 14.166, SD = 1.855), outperformed EG2 (M = 9.277, SD = 4.376), who in turn outstripped CG (M = 3.633, SD = 1.680).

The above table (Table 5) represents a significant difference between conditions on the posttest (df = 2, F = 62.529, *p* = 0.001) with a large effect size (Partial Eta Squared = 0.710).

**Table 5:** Tests of between-subjects effects on planning time’s effect on vocabulary learning on the posttest

Source	Type III sum of squares	df	Mean square	F	Sig.	Partial sta squared
Corrected model	1059.593	2	529.796	62.529	0.000	0.710
Intercept	4302.296	1	4302.296	507.779	0.000	0.909
Group	1059.593	2	529.796	62.529	0.000	0.710
Error	432.111	51	8.473			
Total	5794.000	54				
Corrected total	1491.704	53				

In Table 6, pairwise comparisons show that EG1 outperformed EG2 on the posttest (Mean Difference = 4.889, *p* = 0.001), which, in turn, outstripped the CG (Mean Difference = 5.944, *p* = 0.001).

**Table 6:** Pairwise comparisons of planning time’s effect on vocabulary learning on the posttest

(I) Group	(J) Group	Mean difference (I-J)	Std. Error	Sig.	95% confidence interval for difference	
					Lower bound	Upper bound
EG1	EG2	4.889	0.970	0.000	2.487	7.291
	CG	10.833	0.970	0.000	8.431	13.235
EG2	EG1	-4.889	0.970	0.000	-7.291	-2.487
	CG	5.944	0.970	0.000	3.543	8.346
CG	EG1	-10.833	0.970	0.000	-13.235	-8.431
	EG2	-5.944	0.970	0.000	-8.346	-3.543

### The effect of WM on vocabulary learning

To measure WM’s impact on vocabulary learning, an ANOVA for each time interval is needed. First, the results of the K-S test are presented to ensure the normality of the data.

Based on Table 7, the results of a K-S test show that regarding WM, the data are normally distributed (*p* > 0.05).

**Table 7:** One-sample Kolmogorov-Smirnov test of WM

		WM
N		54
Normal parameters	Mean	3.648
	Std. Deviation	1.760
Most extreme differences	Absolute	0.149
	Positive	0.122
	Negative	− 0.149
Kolmogorov-Smirnov Z		1.095
Asymp. Sig. (2-tailed)		0.181

Table 8 shows that on the pretest EG1 high-WM learners (M = 3.222, SD = 1.653, *N* = 9), EG1 low-WM learners (M = 3.142, SD = 2.340, *N* = 7), EG2 high-WM learners (M = 4.000, SD = 1.658, *N* = 9), EG2 low-WM learners (M = 3.777, SD = 1.922, *N* = 9), CG high-WM learners (M = 3.666, SD = 1.802, *N* = 9), and CG low-WM learners (M = 3.090, SD = 1.972, *N* = 11) performed similarly.

**Table 8:** Descriptive statistics of WM’s effect on the pretest

WM	Mean	Std. Deviation	N
EG1 WM high	3.222	1.563	9
EG1 WM low	3.142	2.340	7
EG2 WM high	4.000	1.658	9
EG2 WM low	3.777	1.922	9
CG WM high	3.666	1.802	9
CG WM low	3.090	1.972	11
Total	3.481	1.819	54

Table 9 indicates that on the pretest, there was no effect for WM (df = 5, F = 0.375, *p* > 0.05).

**Table 9:** Tests of between-subjects effects of WM’s effect on the pretest

Source	Type III sum of squares	df	Mean square	F	Sig.	Partial eta squared
Corrected model	6.604	5	1.321	0.375	0.863	0.038
Intercept	644.089	1	644.089	183.069	0.000	0.792
WM	6.604	5	1.321	0.375	0.863	0.038
Error	168.877	48	3.518			
Total	830.000	54				
Corrected total	175.481	53				

Based on Table 10, EG1 high-WM learners (M = 14.222, SD = 1.855, *N* = 9), and EG1 low-WM learners (M = 14.714, SD = 1.799, *N* = 7) performed similarly on the posttest. In turn, these two outperformed EG2 high-WM learners (M = 11.777, SD = 2.587, *n* = 9), and EG2 low-WM learners (M = 7.222, SD = 3.419, *n* = 9). Furthermore, CG high-WM learners (M = 4.444, SD = 4.901, *n* = 9) also outperformed their low-WM peers (M = 3.636, SD = 1.747, *n* = 11).

**Table 10:** Descriptive statistics of WM’s effect on the posttest

WM	Mean	Std. Deviation	N
EG1 WM high	14.222	1.855	9
EG1 WM low	14.714	1.799	7
EG2 WM high	11.777	2.587	9
EG2 WM low	7.222	3.419	9
CG WM high	4.444	4.901	9
CG WM low	3.636	1.747	11
Total	8.925	5.305	54

According to the above-presented table (Table 11), there was a significant effect for WM on the posttest (df = 5, F = 24.753, *p* = 0.001). The effect size was large (Partial Eta Squared = 0.721).

**Table 11:** Tests of between-subjects effects of WM’s effect on the posttest

Source	Type III sum of squares	df	Mean square	F	Sig.	Partial eta squared
Corrected model	1074.841	5	214.968	24.753	0.000	0.721
Intercept	4626.792	1	4626.792	532.756	0.000	0.917
WM	1074.841	5	214.968	24.753	0.000	0.721
Error	416.863	48	8.685			
Total	5794.000	54				
Corrected total	1491.704	53				

Table 12 shows that on the posttest, there was not a significant difference between EG1 high-WM learners and their low-WM counterparts (Mean Difference = − 0.492, *p* = 0.001). However, EG1 high-WM subjects outperformed EG2 high-WM participants (Mean Difference = 2.444, *p* = 0.001), which, in turn, their outstripped EG2 low-WM peers (Mean Difference = 4.556, *p* = 0.029). Additionally, CG high-WM subjects outperformed their low-WM peers, but in a nonsignificant way (Mean Difference = 0.808, *p* > 0.05).

**Table 12:** Pairwise comparisons of WM’s effect on the posttest

(I) WM	(J) WM	Mean difference (I-J)	Std. Error	Sig.	95% confidence interval for difference	
					Lower bound	Upper bound
EG1 WM high	EG1_WM_low	− 0.492	1.485	1.000	-5.080	4.096
	EG2 WM high	2.444	1.389	1.000	-1.847	6.736
	EG2 WM low	7.000	1.389	0.000	2.708	11.292
	CG WM high	9.778	1.389	0.000	5.486	14.069
	CG WM low	10.586	1.325	0.000	6.494	14.678
EG1 WM low	EG1 WM high	0.492	1.485	1.000	-4.096	5.080
	EG2 WM high	2.937	1.485	0.807	-1.651	7.524
	EG2 WM low	7.492	1.485	0.000	2.904	12.080
	CG WM high	10.270	1.485	0.000	5.682	14.858
	CG WM low	11.078	1.425	0.000	6.676	15.480
EG2 WM high	EG1 WM high	-2.444	1.389	1.000	-6.736	1.847
	EG1 WM low	-2.937	1.485	0.807	-7.524	1.651
	EG2 WM low	4.556	1.389	0.029	0.264	8.847
	CG WM high	7.333	1.389	0.000	3.042	11.625
	CG WM low	8.141	1.325	0.000	4.050	12.233
EG2 WM low	EG1 WM high	-7.000	1.389	0.000	-11.292	-2.708
	EG1 WM low	-7.492	1.485	0.000	-12.080	-2.904
	EG2 WM high	-4.556	1.389	0.029	-8.847	− 0.264
	CG WM high	2.778	1.389	0.768	-1.514	7.069
	CG WM low	3.586	1.325	0.141	− 0.506	7.678
CG WM high	EG1 WM high	-9.778	1.389	0.000	-14.069	-5.486
	EG1 WM low	-10.270	1.485	0.000	-14.858	-5.682
	EG2 WM high	-7.333	1.389	0.000	-11.625	-3.042
	EG2 WM low	-2.778	1.389	0.768	-7.069	1.514
	CG WM low	0.808	1.325	1.000	-3.284	4.900
CG WM low	EG1_WM_high	-10.586	1.325	0.000	-14.678	-6.494
	EG1 WM low	-11.078	1.425	0.000	-15.480	-6.676
	EG2_WM_high	-8.141	1.325	0.000	-12.233	-4.050
	EG2_WM_low	-3.586	1.325	0.141	-7.678	0.506
	CG WM high	− 0.808	1.325	1.000	-4.900	3.284

### The effect of FI/D on vocabulary learning

To assess the influence of FI/D on vocabulary acquisition, it is essential to conduct separate ANOVA analyses for each designated time interval. Initially, the outcomes of the K-S test are provided to ascertain the normal distribution of the data.

The outcomes of a K-S test in Table 13 indicate that, with respect to FI/D, the data exhibit normal distribution (*p* > 0.05).

**Table 13:** One-sample Kolmogorov-Smirnov test of cognitive style

		Cognitive style
N		54
Normal parameters	Mean	3.5000
	Std. Deviation	1.74561
Most extreme differences	Absolute	0.138
	Positive	0.138
	Negative	− 0.138
Kolmogorov-Smirnov Z		1.016
Asymp. Sig. (2-tailed)		0.253

Based on Table 14, EG1 FI subjects (M = 3.222, SD = 1.563, *N* = 9), EG1 FD participants (M = 3.444, SD = 2.242, *N* = 9), EG2 FI ones (M = 3.400, SD = 1.897, *N* = 10), EG2 FD participants (M = 4.250, SD = 1.488, *N* = 8), CG FI learners (M = 3.750, SD = 1.752, *N* = 8), and CG2 FD subjects (M = 3.000, SD = 2.054, *N* = 10) performed more or less the same on the pretest.

**Table 14:** Descriptive statistics of cognitive style on the pretest

Cognitive style	Mean	Std. Deviation	N
EG1 FI	3.222	1.563	9
EG1 FD	3.444	2.242	9
EG2 FI	3.400	1.897	10
EG2 FD	4.250	1.488	8
CG FI	3.750	1.752	8
CG FD	3.000	2.054	10
Total	3.481	1.819	54

Table 15 shows that there was no effect of FI/D on the pretest (df = 5, F = 0.477, *p >* 0.05).

**Table 15:** Tests of between-subjects effects of cognitive style on the pretest

Source	Type III sum of squares	df	Mean square	F	Sig.	Partial eta squared
Corrected model	8.304	5	1.661	0.477	0.792	0.047
Intercept	660.205	1	660.205	189.558	0.000	0.798
Cognitive style	8.304	5	1.661	0.477	0.792	0.047
Error	167.178	48	3.483			
Total	830.000	54				
Corrected total	175.481	53				

Table 16 demonstrates that on the posttest, EG1 FI learners (M = 14.222, SD = 1.855) and EG1 FD participants (M = 14.111, SD = 1.964) performed similarly. However, EG2 FI learners (M = 9.700, SD = 4.164) outperformed EG2 FD subjects (M = 8.750, SD = 4.862). Additionally, CG2 FD (M = 3.600, SD = 1.837) outstripped CG FI ones (M = 3.000, SD = 1.511).

**Table 16:** Descriptive statistics of cognitive style on the posttest

Cognitive style	Mean	Std. Deviation	N
EG1 FI	14.222	1.855	9
EG1 FD	14.111	1.964	9
EG2 FI	9.700	4.164	10
EG2 FD	8.750	4.862	8
CG FI	3.000	1.511	8
CG FD	3.600	1.837	10
Total	8.925	5.305	54

Table 17 reveals that there was a significant effect of cognitive style on the posttest with a very large effect size (df = 5, F = 23.981, *p* = 0.001, Partial Eta Squared = 0.714).

**Table 17:** Tests of between-subjects effects of cognitive style on the posttest

Source	Type III sum of squares	df	Mean square	F	Sig.	Partial eta squared
Corrected model	1065.259^a^	5	213.052	23.981	0.000	0.714
Intercept	4239.343	1	4239.343	477.175	0.000	0.909
Cognitive style	1065.259	5	213.052	23.981	0.000	0.714
Error	426.444	48	8.884			
Total	5794.000	54				
Corrected total	1491.704	53				

Based on the pairwise comparisons in Table 18, EG1 FI learners performed similarly to EG1 FD learners (Mean difference = 0.111, *p* > 0.05). However, EG1 FI learners outperformed EG2 FI ones (Mean Difference = 4.522, *p* < 0.05). Similarly, EG1 FI participants outstripped EG2 FD subjects (Mean Difference = 5.472, *p* < 0.05). Furthermore, EG1 FI learners outperformed CG FI learners (Mean Difference = 11.222, *p* = 0.001), and CG FD participants (Mean Difference = 10.622, *p* = 0.001). EG1 FD learners also outperformed EG2 FI, EG2 FD, CG FI, and CG FD learners as well.

**Table 18:** Pairwise comparisons of cognitive style’s effect on the posttest

(I) Cognitive style	(J) Cognitive style	Mean difference (I-J)	Std. Error	Sig.	95% confidence interval for difference	
					Lower bound	Upper bound
EG1 FI	EG1 FD	0.111	1.405	1.000	-4.230	4.452
	EG2 FI	4.522	1.370	0.027	0.291	8.753
	EG2 FD	5.472	1.448	0.007	0.998	9.946
	CG FI	11.222	1.448	0.000	6.748	15.696
	CG FD	10.622	1.370	0.000	6.391	14.853
EG1 FD	EG1 FI	− 0.111	1.405	1.000	-4.452	4.230
	EG2 FI	4.411	1.370	0.034	0.180	8.642
	EG2 FD	5.361	1.448	0.008	0.887	9.835
	CG FI	11.111	1.448	0.000	6.637	15.585
	CG FD	10.511	1.370	0.000	6.280	14.742
EG2 FI	EG1 FI	-4.522	1.370	0.027	-8.753	− 0.291
	EG1 FD	-4.411	1.370	0.034	-8.642	− 0.180
	EG2 FD	0.950	1.414	1.000	-3.418	5.318
	CG FI	6.700	1.414	0.000	2.332	11.068
	CG FD	6.100	1.333	0.001	1.982	10.218
EG2 FD	EG1 FI	-5.472	1.448	0.007	-9.946	− 0.998
	EG1 FD	-5.361	1.448	0.008	-9.835	− 0.887
	EG2 FI	− 0.950	1.414	1.000	-5.318	3.418
	CG FI	5.750	1.490	0.005	1.146	10.354
	CG FD	5.150	1.414	0.010	0.782	9.518
CG FI	EG1 FI	-11.222	1.448	0.000	-15.696	-6.748
	EG1 FD	-11.111	1.448	0.000	-15.585	-6.637
	EG2 FI	-6.700	1.414	0.000	-11.068	-2.332
	EG2 FD	-5.750	1.490	0.005	-10.354	-1.146
	CG FD	− 0.600	1.414	1.000	-4.968	3.768
CG FD	EG1 FI	-10.622	1.370	0.000	-14.853	-6.391
	EG1 FD	-10.511	1.370	0.000	-14.742	-6.280
	EG2 FI	-6.100	1.333	0.001	-10.218	-1.982
	EG2 FD	-5.150	1.414	0.010	-9.518	− 0.782
	CG FI	0.600	1.414	1.000	-3.768	4.968

In short, the study investigated the impact of planning time, WM, and cognitive styles, specifically FI/D, on vocabulary learning outcomes. The analysis employed ANOVA for each time interval, supported by the K-S test to ensure data normality. For planning time, the results showed that the experimental group with planning time (EG1) significantly outperformed both the experimental group without planning time (EG2) and the CG on the posttest, highlighting a substantial difference between conditions (*p* = 0.001, Partial Eta Squared = 0.710). Similarly, WM exhibited a significant effect on the posttest, with high-WM learners outperforming low-WM counterparts across groups (*p* = 0.001, Partial Eta Squared = 0.721). The influence of FI/D on vocabulary learning also yielded significant effects, with FI learners outperforming FD learners on the posttest (*p* = 0.001, Partial Eta Squared = 0.714). Pairwise comparisons revealed nuanced differences, emphasizing the intricate interplay of planning time, WM, and cognitive styles in shaping vocabulary acquisition outcomes.

## Discussion

The results clearly indicate that providing planning time before engaging in communicative tasks has a substantial positive impact on vocabulary acquisition. Learners who received planning time outperformed their counterparts who did not, as well as those in the control group. This underscores the importance of strategic planning in language learning contexts, allowing learners to organize their thoughts and access relevant vocabulary more effectively during communicative tasks. Educators and curriculum designers should consider incorporating such planning phases into language instruction to optimize vocabulary learning outcomes.

The significant influence of WM on vocabulary acquisition reaffirms the well-established link between cognitive processes and language learning. High-WM learners consistently outperformed their low-WM counterparts across all groups on the posttest. This underscores the need for instructional strategies that accommodate learners with varying WM capacities. Future research could explore specific interventions or instructional designs tailored to enhance vocabulary acquisition for learners with lower WM capacities.

Moreover, the study illuminates the impact of FI/D on vocabulary learning, with FI learners exhibiting superior performance on the posttest. This finding aligns with the broader literature on cognitive styles and language learning, emphasizing the need for personalized approaches that consider individual differences in cognitive processing. Educators should be attuned to learners’ cognitive styles, potentially tailoring instructional methods to better suit their cognitive preferences.

The obtained results can be attributed to the interplay of cognitive processes and instructional interventions in the language learning context. The observed positive effect of planning time on vocabulary acquisition aligns with cognitive theories emphasizing the significance of pre-task planning in facilitating information processing and retrieval. The opportunity for learners to strategize and organize their thoughts before engaging in communicative tasks likely contributed to a more efficient encoding of new vocabulary. Additionally, the consistent superiority of high-WM learners suggests that individuals with greater WM capacity possess enhanced cognitive resources for managing linguistic information, thereby exhibiting better retention and recall. The positive impact of FI cognitive styles on vocabulary learning may be linked to a greater ability to discern and focus on specific linguistic details, fostering a more analytical approach to word acquisition. Overall, these results underscore the intricate interplay between cognitive factors and instructional methodologies, providing valuable insights for educators seeking to tailor language instruction to learners’ cognitive profiles and optimize vocabulary acquisition outcomes.

The theoretical contributions of this study lie in its nuanced exploration of language learning dynamics, specifically delving into the roles of planning time, WM, and FI/D within the framework of TBLT. By incorporating planning time as a strategic element in language tasks, the study extends existing TBLT principles, emphasizing its potential to enhance vocabulary acquisition and overall language proficiency. The examination of cognitive styles, particularly field independence, contributes to a deeper understanding of how individual differences impact language learning. The study challenges existing frameworks by highlighting the need for tailored instructional approaches that recognize and accommodate diverse cognitive processing styles. Moreover, the emphasis on working memory’s multifaceted contributions to vocabulary acquisition adds a novel dimension to theoretical discussions on cognitive processes in language learning. This study thus enriches and extends current theoretical frameworks in the field of language acquisition, offering valuable insights for researchers, educators, and curriculum designers.

The studies presented in the empirical background share a common focus on the effectiveness of various instructional methodologies in enhancing vocabulary learning and language performance among EFL learners. Notably, the findings of our study align with the positive outcomes observed in [31], where CALL with TBLT significantly outperformed traditional instruction in both receptive and productive vocabulary posttests. Our emphasis on pre-task planning time resonates with [34], revealing that providing planning time for second-language speech tasks led to significantly better performance in terms of fluency, accuracy, and complexity. Additionally, our investigation into the influence of WM on vocabulary learning corresponds to [37], which explored the role of WM in multimedia vocabulary learning and found that complex and phonological WM significantly contributed to vocabulary acquisition.

In contrast, our study diverges from [32] and [33] in terms of the instructional interventions used. While (32) incorporated the FlipGrid application for technology-mediated TBLT in descriptive writing, and [33] implemented TBLT methods with a focus on word games for Chinese EFL students, our study examined the impact of planning time and cognitive styles on vocabulary learning. Our findings also differ from [35], which explored the effects of pre-task planning on Chinese EFL learners’ selection of referential expressions in oral narratives. Our study delved into the broader aspects of vocabulary acquisition, considering the effects of planning time and cognitive styles on overall vocabulary learning rather than specific discourse elements.

Moreover, our research extends beyond the traditional focus on task performance in TBLT, as demonstrated in [36], which explored the intricacies of collaborative pre-task planning among Japanese university learners. While [36] focused on the planning processes and task performances themselves, our study explored the impact of planning time and cognitive styles on vocabulary learning outcomes.

The studies investigating incidental vocabulary learning, such as [39], explored different factors like video types and repetition, which were not within the scope of our study. Additionally, cognitive styles, specifically field independence, have been addressed in [41], emphasizing their influence on the efficacy of processing instruction. This contrasts with our study, where cognitive styles were considered in the context of pre-task planning time.

In the context of TBLT, our study aligns with the core principles outlined by [17], emphasizing the importance of communicative tasks in language education. The incorporation of planning time in our investigation resonates with the TBLT framework proposed by [21], which includes stages such as pre-task, task cycle, and language focus. By introducing planning time as a crucial factor in vocabulary learning, our study extends the TBLT model to highlight its potential impact on language proficiency development.

The concept of planning time finds theoretical roots in cognitive and psycholinguistic frameworks, particularly IPM and CRT. Our study supports these models by demonstrating that planning time serves as a dedicated phase for learners to engage in metacognitive processes, strategically allocate cognitive resources, and enhance the quality of language production. This aligns with the cognitive rehearsal mechanism proposed by the CRT, suggesting that planning time contributes to the effective use of WM and the integration of new vocabulary into language production.

The role of WM in language learning is central to our study’s findings. As per Baddeley’s model, WM is engaged during language production, aiding in the selection and arrangement of words within sentences. Our study contributes to this understanding by highlighting the multifaceted influence of WM on vocabulary acquisition. The findings underscore that WM enables the temporary storage of new words and plays a vital role in sentence comprehension, parsing, and interpretation of complex linguistic structures.

Additionally, our study considers the cognitive style of FI/D in the context of language learning. By exploring how individual differences in cognitive styles, such as field independence, interact with planning time and impact vocabulary learning outcomes, our research extends the theoretical understanding of cognitive styles in language education. This aligns with Witkin’s theory, emphasizing that individuals exhibit consistent preferences in how they approach and organize information, with potential implications for language learning strategies.

The study’s implications for language teachers are significant. The findings highlight the importance of incorporating planning time into language lessons, particularly during vocabulary learning tasks. Teachers should recognize planning time as a valuable phase that allows learners to engage in metacognitive processes and strategically allocate cognitive resources. Implementing structured planning activities can enhance students’ vocabulary acquisition and overall language proficiency. Additionally, understanding the influence of individual cognitive styles, such as field independence, can guide teachers in tailoring instructional strategies to meet the diverse learning needs of students. Acknowledging the role of WM in language tasks also emphasizes the need for instructional approaches that support the efficient processing and integration of new vocabulary. Educators can seamlessly integrate planning time into language instruction by adopting a structured and purposeful approach. First, they can incorporate pre-task planning phases within lesson plans, allocating dedicated time for learners to strategize and organize their thoughts before engaging in communicative tasks. Providing clear instructions and guidance on effective planning strategies can empower students to make optimal use of this time. Educators may also introduce planning templates or checklists to scaffold learners in organizing their ideas. Additionally, fostering a supportive classroom environment that encourages collaboration during the planning phase can enhance the effectiveness of this strategy. Regularly reflecting on the outcomes of planning time and adjusting instructional approaches based on student feedback can further refine the implementation of this integration, ensuring that it aligns seamlessly with the broader language learning objectives.

For materials developers, the study suggests designing language learning materials that explicitly incorporate planning time elements. Learning resources should include tasks that require learners to engage in strategic planning before executing language production. Integrating multimedia and interactive components can further enhance the effectiveness of materials, considering the role of WM in multimedia learning. Materials should also be adaptable to accommodate diverse cognitive styles, providing learners with opportunities to approach language tasks in ways that align with their individual preferences. This tailored approach can contribute to more inclusive and effective language learning materials. For materials developers, this study suggests a transformative approach in crafting language learning materials that align with the cognitive processes identified in the research. The intentional inclusion of planning time within instructional materials emerges as a crucial consideration, recognizing its potential to enhance vocabulary acquisition and overall language proficiency. Developers should design materials that offer strategic language planning opportunities within tasks, striking a balance between meaning-focused activities and chances for learners to strategically organize their language output. Furthermore, acknowledging and accommodating individual cognitive styles, especially field independence, should guide the creation of materials tailored to diverse learning needs. The incorporation of these insights into instructional materials can contribute to more inclusive, engaging, and effective language education.

Syllabus designers can use the study’s insights to inform the structure and content of language education programs. Emphasizing the integration of planning time as a dedicated phase within TBLT can enhance syllabi. The study underscores the need for a balanced approach that prioritizes meaning in tasks but also allocates time for language form considerations. Syllabi should account for the diverse cognitive styles of learners, recognizing that preferences for detail-oriented or context-dependent processing may influence language learning outcomes. Additionally, syllabus designers can explore ways to scaffold and support WM demands in language tasks, ensuring that instructional sequences align with cognitive processing capacities. Syllabus designers can draw valuable insights from this study to inform the development of language education curricula. The intentional integration of planning time into the syllabus can be emphasized, recognizing its potential to positively impact vocabulary acquisition and language proficiency. Designers may consider incorporating tasks that provide learners with strategic language planning opportunities, aligning with the principles of TBLT. Additionally, understanding and addressing individual differences in cognitive styles, particularly field independence, should influence syllabus design to create learning environments that cater to diverse learner preferences. By infusing these considerations into syllabi, designers contribute to the creation of learner-centered curricula that optimize language learning outcomes and cater to the cognitive intricacies of language acquisition.

Curriculum developers can use the study’s implications to refine language education curricula. Integrating planning time as a core component within the curriculum can contribute to more effective vocabulary learning outcomes. The study emphasizes the dynamic interplay between task complexity, cognitive styles, and WM, suggesting that curricula should consider these factors when designing language learning pathways. Providing flexibility in instructional approaches to accommodate different cognitive styles ensures that the curriculum meets the diverse needs of learners. Curriculum developers can also explore ways to integrate technology and multimedia resources that align with the findings on WM and vocabulary learning.

Policymakers in the education sector can leverage the study’s implications to shape language education policies. Recognizing the role of planning time in language tasks, policymakers can encourage the integration of task-based approaches in language education frameworks. Policies that support professional development for teachers, specifically focusing on the implementation of planning time strategies, can enhance the overall quality of language instruction. Consideration of individual differences, such as cognitive styles, should inform policies aimed at promoting inclusive language education. Policymakers can also support research initiatives that further explore the intersection of cognitive processes and language learning, fostering evidence-based practices in language education policy. Policymakers can play a pivotal role in promoting the integration of planning time into language education by implementing targeted changes at the institutional level. Firstly, they can advocate for the inclusion of planning time guidelines in language curriculum frameworks, emphasizing its importance as a cognitive preparation phase for language tasks. Policymakers may also allocate resources for teacher training programs that focus on effective strategies for integrating planning time into instructional practices. Additionally, incorporating planning time considerations into standardized assessments can signal its significance and encourage educators to prioritize this element in their teaching. Policymakers could collaborate with educational researchers to explore the impact of planning time on language learning outcomes, providing evidence-based insights to inform policy decisions. Finally, fostering a culture of flexibility and experimentation in language education policies can create an environment where educators feel empowered to explore and implement innovative approaches, including the strategic integration of planning time.

## Conclusion

In conclusion, this study delved into the intricate dynamics of language learning, specifically focusing on the roles of planning time, WM, and cognitive styles. Through a meticulous examination of empirical data, the study unveiled valuable insights that contribute to our understanding of effective language instruction. The incorporation of planning time in language tasks emerged as a crucial factor, showcasing its potential to enhance vocabulary acquisition and overall language proficiency. The study underscored the importance of striking a balance between meaning-focused tasks and opportunities for strategic language planning, aligning with the principles of TBLT.

Moreover, the exploration of cognitive styles, particularly field independence, emphasized the need for tailored instructional approaches. Recognizing and accommodating individual differences in cognitive processing styles can lead to more inclusive and effective language education. The study shed light on how learners with different cognitive styles may approach language tasks, influencing their strengths and preferences. This nuanced understanding provides educators and curriculum designers with valuable insights to create learning environments that cater to diverse learning needs.

The role of WM in language tasks was another pivotal aspect addressed in this study. Understanding the multifaceted contributions of WM to vocabulary acquisition, comprehension, and production highlighted its significance in language learning. The findings reinforced the idea that supporting learners in managing cognitive resources, especially through strategic planning, positively impacts language performance.

As language education continues to evolve, these findings carry practical implications for language teachers, materials developers, syllabus designers, curriculum developers, and policymakers. The study advocates for the intentional integration of planning time into language lessons, the development of materials that align with cognitive processes, and the consideration of individual cognitive styles in curriculum and policy decisions. By embracing these insights, educators and policymakers can contribute to more effective, inclusive, and learner-centered language education practices. This study thus adds a nuanced layer to the ongoing discourse on optimizing language learning experiences and outcomes.

The study has certain limitations that warrant consideration. Firstly, the sample used in the research was relatively homogeneous, consisting of participants with similar demographic characteristics. As a result, the findings may not be fully generalizable to a more diverse population of language learners. Future research could benefit from including a broader range of participants, considering factors such as age, language proficiency, and cultural background to enhance the external validity of the results. Additionally, the study focused on specific language tasks, potentially limiting the generalizability of the findings to a broader spectrum of language learning activities. Exploring a more extensive array of tasks and contexts could offer a more nuanced understanding of how planning time, WM, and cognitive styles operate across various linguistic activities. Moreover, the study predominantly concentrated on short-term outcomes, and investigating the long-term effects of planning time and cognitive styles on language learning could provide valuable insights into the sustainability of these effects.

In terms of suggestions for further research, a cross-cultural exploration could enhance the generalizability of findings. Including participants from diverse cultural and linguistic backgrounds in future studies may reveal variations in the impact of planning time, WM, and cognitive styles on language learning. Longitudinal studies tracking learners over an extended period could contribute valuable insights into the enduring effects of instructional interventions and cognitive processes on language proficiency development. Moreover, as technology continues to play a significant role in language education, future research could explore the interplay between digital tools, planning time, and cognitive styles. Understanding how technology influences language learning dynamics can inform the design of more effective and engaging language learning environments. Further investigations into the impact of task complexity on planning time and cognitive processes could deepen our understanding of optimal conditions for language learning. Research exploring how learners navigate planning and memory resources in response to varying task complexities would provide practical guidance for educators. Finally, building on the findings related to cognitive styles, researchers could delve into the development of inclusive pedagogical strategies that cater to learners with different cognitive preferences. Tailoring instructional approaches based on cognitive styles could contribute to more personalized and effective language education.

As another suggestion for further research, future researchers may consider delving into the dynamic intersection of digital tools with planning time and cognitive processes, thereby introducing a forward-looking dimension to their study. With the pervasive influence of technology in modern education, investigating how digital tools interact with the planning time and cognitive mechanisms explored in the current study could yield valuable insights. Examining the impact of technology on language learning dynamics, specifically in the context of planning time and cognitive styles, may contribute to a more comprehensive understanding of effective instructional strategies in technologically mediated learning environments. This forward-looking exploration has the potential to inform educators and policymakers on leveraging digital tools for enhanced language learning outcomes.

Future research in the field should prioritize key areas identified in this study to make a substantial impact. Firstly, exploring cross-cultural variations in the impact of planning time, WM, and cognitive styles on language learning could enhance the generalizability of findings. Longitudinal studies tracking learners over time would provide insights into the enduring effects of instructional interventions and cognitive processes on language proficiency development. Additionally, investigating the interplay between digital tools, planning time, and cognitive styles in the context of language education can inform the design of more effective and engaging learning environments. Further research into the impact of task complexity on planning time and cognitive processes would deepen our understanding of optimal conditions for language learning. Lastly, delving into the development of inclusive pedagogical strategies tailored to learners with different cognitive preferences could contribute to more personalized and effective language education. Prioritizing these key areas ensures that future research addresses critical gaps and advances the field in a meaningful way.

Alternative explanations for the observed results could be considered to ensure a comprehensive interpretation of the findings. Firstly, individual differences among participants, such as varying levels of motivation, learning styles, or prior exposure to task-based language teaching, might have influenced the outcomes. Additionally, the specific characteristics of the communicative tasks or the duration of the study could be contributing factors. It’s plausible that certain unaccounted variables, external to the experimental design, may have influenced vocabulary acquisition, warranting further exploration. Moreover, the homogeneity of the participant population in terms of linguistic background and educational experiences may limit the generalizability of the results to more diverse language learner cohorts. Considering these alternative explanations allows for a nuanced understanding of the study’s outcomes and points towards potential areas for refinement or future investigations.

In conclusion, while this study offers valuable insights into the roles of planning time, WM, and cognitive styles in language learning, it is essential to acknowledge its limitations and consider avenues for further research. Addressing these limitations and pursuing the suggested research directions can contribute to a more comprehensive understanding of the factors influencing language learning outcomes and inform the development of more effective language teaching practices.

## Data Availability

The dataset of the present study is available upon request from the corresponding author.
